# Lifespan risks of growing up in a family with mental illness or substance abuse

**DOI:** 10.1038/s41598-020-72064-w

**Published:** 2020-09-22

**Authors:** Vera Clemens, Oliver Berthold, Andreas Witt, Cedric Sachser, Elmar Brähler, Paul L. Plener, Bernhard Strauß, Jörg M. Fegert

**Affiliations:** 1 Department of Child and Adolescent Psychiatry/Psychotherapy, University of Ulm, Steinhövelstr. 5, 89073 Ulm, Germany; 2 Department for Psychosomatic Medicine and Psychotherapy, University Medical Center of Johannes Gutenberg University of Mainz, Mainz, Germany; 3 Department of Medical Psychology and Medical Sociology, University of Leipzig, Leipzig, Germany; 4 Department for Child and Adolescent Psychiatry, Medical University of Vienna, Vienna, Austria; 5 Institute of Psychosocial Medicine and Psychotherapy, Jena University Hospital, Jena, Germany

**Keywords:** Risk factors, Epidemiology, Paediatric research

## Abstract

Growing up in a family with one member being affected by mental health problems or substance abuse is an adverse childhood experience which can lead to socioeconomic and health-related impairments in later life. Furthermore, the risk of child maltreatment is increased in affected families, which often adds to the individual risk factors. However, the interdependence between the particular risk factors is not well understood. To examine the correlation between mental health problems or substance abuse and child maltreatment within families and long term consequences for affected children, a cross sectional population representative survey in Germany (N = 2,531) has been conducted. The risk of child maltreatment was 5 to 5.6 times higher if mental illness and 4.9 to 6.9 times higher if substance abuse of a family member was reported. Furthermore, the risk of health problems, including obesity, decreased life satisfaction, lower income, low educational achievement, unemployment and living without a partner was increased if participants grew up in a family affected by mental health problems or substance abuse. All associations were mediated significantly by child maltreatment. These results point towards an urgent need for greater awareness for child protection issues in families affected by mental health problems or substance abuse.

## Introduction

Adverse childhood experiences (ACE) can affect life in different ways. The long-term consequences are widespread and can include psychosocial and economic impairments, a significant reduction in quality of life and increased morbidity due to both, mental and somatic health problems^1^. In his famous ACE study 20 years ago, Vincent Felitti demonstrated that with increasing number of ACEs the risk of suicide attempts, smoking, alcohol and drug consumption and many diseases increased up to 12-fold^2^. Recently published studies on population representative samples in Germany and the U.S. show that child maltreatment is associated with an increased risk for leading causes of morbidity and mortality, including cardiovascular and oncological diseases^3,4^. Overall, ACEs can lead to a shortening of the life span of up to 20 years^5^. In addition to these serious consequences for the life of each individual affected, they lead to an enormous economic burden with annual expenditures between 11 and 30 billion euros in Germany alone^6^. Taken together, ACEs can be considered as major public health problems.

ACEs include all forms of child maltreatment, defined as any intentional acts of commission or omission that cause harm or expose the child/adolescent to the risk of harm—regardless of the intention to harm^7^. Child maltreatment is frequent—a recent epidemiologic study from a German sample reports at least moderate experience of emotional abuse in 6.6%, physical abuse in 6.7%, sexual abuse in 7.6% and emotional and physical neglect in 13.3% and 22.5%, respectively^8^. The term ACE also includes stressful childhood experiences such as intimate partner violence between parents, parental separation or divorce, and being part of a family affected by mental health problems or substance abuse. The last two items are of particular relevance to mental health professionals, as 10–30% of inpatients with psychiatric disorders have underage children^9,10^. A better understanding of the possible long-term consequences of growing up in families affected by mental health problems or substance abuse is essential for effective prevention.

Earlier research showed that families affected by mental health problems or substance abuse are at higher risk for child maltreatment^11–13^. As shown above, child maltreatment is a powerful risk factor on its own^1,3,14,15^. For effective prevention, we need to better understand the interplay between these risk factors, namely growing up in a family affected by mental health problems and substance abuse, and child maltreatment. In a previous analysis, we were able to show that child maltreatment mediates the risk of household dysfunction for depression, anxiety, life quality and overall health status^16^. Based on these associations, we hypothesized that child maltreatment partly mediates the long term consequences of growing up in a family affected by mental health problems and substance abuse.

Therefore, we examined health and socio-economic indicators in adulthood as well as the experience of child maltreatment in dependence of growing up in a family affected by mental health problems or substance abuse in a sample of the German population representative in regard of gender and age. A mediation analysis for child maltreatment in relation to growing up in a family affected by mental health problems or substance abuse.

## Methods

A representative sample of the German population was randomly collected by a social research institute (USUMA, Berlin, Germany) as described elsewhere^17^.

Data collection took place between November 2017 and February 2018. The sample was representative of the German population over 14 years of age in terms of age and gender. Out of 5,160 identified addresses, 2,531 persons participated in the study (response rate: 49.1%).

All participants received information and written consent. In the case of minors, the participants gave their consent after clarification with the consent of their caregivers. The study was conducted in accordance with the Declaration of Helsinki and complied with the ethical guidelines of the International Code of Marketing and Social Research Practice of the International Chamber of Commerce and the European Society of Opinion and Marketing Research. The study was approved by the Ethics Commission of the Medical Faculty of the University of Leipzig.

### Measures

The prevalence of different forms of ACEs was assessed using the German version of the Adverse Childhood Experiences Questionnaire, a standard tool for retrospective assessment of ACEs with satisfactory internal consistency (Cronbachs α = 0.76)^18^. The questions relating growing up in a family affected by mental health problems or substance abuse were used as independent variables in all analyses. The wording of the questionswas: "Did you live with someone who had alcohol problems, was addicted to alcohol or drugs?" and "Did a member of your household suffer from depression, mental health problems or did attempt suicide?”.

Different psychosocial and health-related variables served as outcomes. In detail, satisfaction with life was assessed based on the question "How satisfied are you at present, all in all, with your life", scale 1 (not satisfied at all) to 11 (completely satisfied) according to Beierlein and colleagues^19^. The state of health was assessed using the EuroQOL 5-Dimensions 3-Level (EQ-5D-3L) questionnaire. The EQ-5D-3L covers five dimensions on current problems: "Mobility", "Self-care", “Usual Activities", "Pain/Physical discomfort" and "Anxiety/Depression". The answers encompass 3 levels: no problems, moderate problems, or severe problems^20^. For the EQ-5D-3L index, all 5 dimesions can be summarized using a formula based on country-specific weights for the individual items^21^. In the present study, weights were used which were derived from a large representative German population sample^22^. Only valid answers were included into the respective analysis. The number of included cases is given for each analysis. The participants were on average 48.6 years old (SD = 18.0), 56.4% were women. 96% stated that they had German citizenship. The sample was representative of the German population over the age of 14 in terms of age and gender. Sample characteristics are presented in Table 1.

**Table 1:** Sample characteristics.

	Total (N = 2,531)
Age (M, SD)	48.6 (18.0)
**Gender (n, %)**	
Female	1,401 (55.4)
Male	1,130 (44.6)
German citizenship (n, %)	2,429 (96.0)
Living with a partner (n, %)	1,351 (53.4)
Number of periods of unemployment (lifetime) (M, SD)	0.79 (1.51)
Monthly household income in € (M, SD)	1759.56 (715.25)
Life satisfaction (M, SD)	8.26 (2.07)
EQ-5D-3L index (M, SD)	0.92 (0.14)
**Highest level of education (n, %)**	
Left school before graduation	56 (2.3)
School graduation	2,186 (75.8)
German baccalaureate	307 (12.5)
University degree	233 (9.5)
**Adverse childhood experiences (ACEs) (n, %)**	
Emotional abuse	316 (12.5)
Physical abuse	230 (9.1)
Sexual abuse	109 (4.3)
Emotional neglect	338 (13.4)
Physical neglect	109 (4.3)
Substance abuse of a family member	421 (16.6)
Mental illness of a family member	267 (10.5)

Presented as mean (M) ± standard deviation (SD) or number of participants (%).

### Statistical analyses

All statistical analyses were performed with SPSS version 21. *p* values correspond to two-tailed tests. The prevalence rates were determined using descriptive analyses. Binary logistic regression analyses were performed in order to analyze the odds for the assessed long-term outcomes if mental illness or substance abuse were present in the family of origin. Age, gender and educational achievement were included into the analyses as co-variates. If school qualification served as outcome of regression analysis, no educational achievement was not included as confounder. Bonferroni corrections for multiple comparisons were performed to guard against type I error inflation and α-levels were adjusted accordingly.

The mediation analyses were carried out with the Hayes PROCESS macro^23^. Path analyses were performed with 5,000 bootstrapping samples. The number of different ACE subtypes (0–5) served as a mediation variable. As potential confounders, age, gender and educational achievement were included into the analyses.

## Results

In total, 267 (10.5%) and 421 (16.6%) of participants reported growing up in a family affected by mental health problems, or substance abuse, respectively. 316 (12.5%) of the participants reported emotional and 230 (9.1%) physical abuse, 109 (4.3%) sexual abuse, 338 (13.4%) emotional and 109 (4.3%) physical neglect (see Table 1).

### Child maltreatment is more frequent in families affected by mental health problems or substance abuse

There was a positive association between experiences of maltreatment and growing up in a family affected by mental health problems or substance abuse. Mental health problems in the family was associated with an increased risk for all subtypes of child maltreatment (ORs 5.07–5.63), as was substance abuse (ORs 4.87–6.91). For growing up in a family affected by substance abuse, the risk of physical abuse (OR 6.81) and physical neglect (OR 6.91) increased the most. If growing up in a family affected by mental health problemswas reported, the risk of emotional abuse (OR 5.63) was highest (see Table 2).

**Table 2:** Frequency and risk of child abuse depending on growing up in a family affected by mental health problems or substance abuse.

	Emotional abuse	Physical abuse	Sexual abuse	Emotional neglect	Physical neglect
**Substance abuse**					
OR (95% CI)	5.91 (4.55;7.69)	6.81 (5.07;9.13)	4.87 (3.26;7.27)	5.06 (3.92;6.53)	6.91 (4.61;10.35)
Wald	176.86	162.44	59.89	155.34	87.64
Nagelkerkes R^2^	0.14	0.16	0.12	0.12	0.14
*p* value	< 0.001	< 0.001	< 0.001	< 0.001	< 0.001
N	2,445	2,441	2,442	2,442	2,443
**Mental health problems**					
OR (95% CI)	5.63 (4.20;7.54)	5.07 (3.67;7.01)	5.27 (3.44;8.08)	5.13 (3.84;6.84)	5.22 (3.40;8.00)
Wald	133.54	96.69	58.07	123.32	57.09
Nagelkerkes R^2^	0.11	0.10	0.11	0.10	0.09
*p* value	< 0.001	< 0.001	< 0.001	< 0.001	< 0.001
N	2,432	2,428	2,430	2,431	2,431

Presented as OR (odds ratio) adjusted for gender, age and educational achievement. Bonferroni correction was performed, *p* < 0.01 was considered as statistically significant. 95% CI = 95% confidence interval.

### Higher risk of poor health and low socioeconomic status in families affected by mental health problems illness or substance abuse

There was a positive correlation between growing up in a family affected by mental health problems or substance abuse and poorer health, obesity and lower socioeconomic status.

Participants were more than twice as likely to be dissatisfied with their lives if the grew up in a family affected by mental health problems or substance abuse. Furthermore there was a positive correlation with not living with a partner, having been unemployed at least once in a lifetime and lower income. Only growing up in a family affected by substance abuse was positively related with low educational achievement (see Table 3).

**Table 3:** Frequency and risk of health and socio-economic impairments depending on living with household members who were substance abusers or mentally ill before the age of 18, adjusted for gender, age and educational achievement.

	Poor Health (< 25. Percentile.)	Low life satisfaction (≤ 7)	Low educational achievement	Not living with a partner	At least once unemployed	Monthly equalized income below 1,250 €	Obesity
**Substance abuse**							
OR (95% CI)	2.58 (1.93;3.46)	2.26 (1.76;2.91)	2.50 (1.42;4.42)	1.45 (1.17;1.79)	2.34 (1.86;2.95)	1.83 (1.46;2.30)	1.49 (1.12;1.97)
*p* value	< 0.001	< 0.001	0.002	0.001	< 0.001	< 0.001	0.006
Nagelkerkes R^2^	0.25	0.05	0.03	0.01	0.07	0.07	0.03
N	2,375	2,390	2,453	2,439	2,298	2,387	2,505
**Mental health problems**							
OR (95% CI)	2.66 (2.08;3.41)	2.82 (2.12;3.76)	1.60 (0.77;3.30)	1.57 (1.21;2.04)	1.58 (1.21;2.07)	1.92 (1.46;2.54)	1.42 (1.01;1.98)
*p* value	< 0.001	< 0.001	0.207 (n.s.)	0.001	0.001	< 0.001	0.042 (n.s.)
Nagelkerkes R^2^	0.26	0.05	0.02	0.01	0.05	0.06	0.03
N	2,364	2,377	2,440	2,426	2,287	2,374	2,492

Bonferroni correction was performed, *p* < 0.007 was considered as statistically significant. Presented as OR (odds ratio). 95% CI = 95% confidence interval.

### Child maltreatment mediates risk for poorer health and lower socioeconomic status

Child maltreatment partially mediated the association between growing up in a family affected by mental health problems and general health, life satisfaction and income in adulthood. The increased risk of more episodes of unemployment in adulthood was mediated fully by experiences of child maltreatment.

The association between growing up in a family affected by mental health problems or substance abuse and general health, life satisfaction, income and unemployment in adulthood is partly mediated by child maltreatment. Linear regression analyses showed no significant association between growing up in a family affected by mental health problems or substance abuse and BMI. Therefore, no mediation analysis was performed (see Table 4).

**Table 4:** Association between growing up in a family affected by mental health problems or substance abuse, child abuse and impairments in adulthood.

	Total effect	Direct effect	Indirect effect (mediation)	% of total effect is mediated
**Mental illness**				
Health (N = 2,344)	− 0.05 (− 0.07; − 0.03)	− 0.03 (− 0.05; − 0.01)	− 0.02 (− 0.03; − 0.0)	40
Life satisfaction (N = 2,350)	− 1.16 (− 1.42; − 0.90)	− 0.77 (− 1.05; − 0.50)	− 0.39 (− 0.52; − 0.28)	34
Income (N = 2,348)	− 191.30 (− 281.93; − 100.66)	− 120.35 (− 214.39; − 25.68)	− 71.26 (− 101.18; − 43.98)	37
Unemployment (N = 2,411)	0.31 (0.12;0.51)	0.11 (− 0.10;0.31)	0.11 (0.13;0.29)	35
BMI (N = 2,394)	n.s	n.s	0.31 (0.94;0.56)	–
**Substance abuse**				
Health (N = 2,335)	− 0.05 (− 0.07; − 0.03)	− 0.03 (− 0.05; − 0.02)	− 0.02 (− 0.03; − 0.01)	40
Life satisfaction (N = 2,361)	− 0.93 (− 1.15; − 0.71)	− 0.55 (− 0.78; − 0.32)	− 0.38 (− 0.50; − 0.27)	41
Income (N = 2,359)	− 214.20 (− 288.03; − 140.37)	− 152.81 (− 231.66; − 73.95)	− 61.39 (− 88.53; − 34.64)	29
Unemployment (N = 2,422)	0.52 (0.36;0.68)	0.36 (0.19;0.53)	0.16 (0.09;0.24)	31
BMI (N = 2,406)	n.s.	n.s.	0.32 (0.10;0.55)	–

Mediation analyses of associations between growing up in a family affected by mental health problems or substance abuse and general health (via EQ-5D-3L index), life satisfaction, normalized income and the frequency of unemployment. Total effect = association between growing up in a family affected by mental health problems or substance abuse and a particular outcome variable without exclusion of child maltreatment. Direct effect = association between growing up in a family affected by mental health problems or substance abuse and a particular outcome variable after exclusion of child maltreatment. Indirect effect = association between the number of experienced forms of child maltreatment with the respective outcome variable. Presented as ß-coefficient and 95% confidence interval, adjusted for gender, age and educational achievement. Bonferroni correction was performed, *p* < 0.01 was considered as statistically significant. Results are siginifcant unless stated otherwise.

## Discussion

This study examined long-term effects on health and socioeconomic status in adulthood related to growing up in a family affected by mental health problems or substance abuse in a sample that is representative for the German population in terms of age and gender. The results demonstrate increased risks for several socioeconomic, psychosocial and health-related problems of affected individuals. Furthermore, for the first time we were able to show a significant part of these effects are mediated by child maltreatment. These associations remained significant after adjustment for relevant sociodemographic confounders. These results suggest that the awareness of child maltreatment should be important issue in the treatment of adults with mental health problems or substance abuse.

The present analysis shows strongly increased risks for all forms of child maltreatment in families affected by mental health problems or substance abuse even after controlling for age, gender, and educational achievement as potential confounders. This is consistent with the results of other studies^11,16,24–26^.

In addition, participants who grew up in a family affected by mental health problems or substance abuse, showed extensive health impairments in adulthood. While the increased risk for mental health problems is known^23^, little literature exists on the consequences for physical health. However, in a study from the U.S. similar to this one, an increased risk for obesity was seen^4^.

While it was already known that children of mentally ill parents have a decreased quality of life^27^ and poorer health^28^, we were now able to show that the reduction in life satisfaction persists well into adulthood in a large representative sample. Nevertheless, the association of ACEs in general and reduced quality of life^29^, reduced mental and physical health is widely known and well assessed^2,30^—but these studies mainly assess the cumulative effect of ACEs or focus on child maltreatment^1^, not assessing the effects of single household dysfunctions.

In our study, growing up in a family affected by mental health problems or substance abuses represents a considerable risk in various socio-economic areas: on average, participants stated more frequently that they had low levels of education and income. Interestingly, the latter even after controlling for level of education. This is consistent with results from earlier studies, which demonstrated a link between the mother's mental illness and lower school grades or later public transfer payments^31,32^.

The associations between growing up in a family affected by mental health problems or substance abuse and long-term outcomes were all mediated by child maltreatment. Depending on the analysis, 29–41% of the strength of the relationships presented was mediated by child maltreatment—in the case of growing up in a family affected by mental health problems and later episodes of unemployment, the relationship was even completely mediated by child maltreatment.

In summary, this data suggests that in order for effective prevention of many long lasting negative effects to the live of affected children, the detection of and intervention to maltreatment in families affected by mental health problems or substance abuse are necessary. Therefore the issue of child maltreatment in the treatment of adult patients for mental health problems or substance abuse must be addressed comprehensively. This could be achieved by interdisciplinary approaches in healthcare institutions treating patients for mental health problems or substance abuse.

However, the mediation analysis also shows that the increased risk to health and socioeconomic status is only partly mediated by child maltreatment—there seem to be other factors that may also be relevant. Low socioeconomic status, social isolation and stigmatization due to mental health problems or substance abuse are both risk factors for, but also consequences of ACEs^33,34^ and thus forming a vicious circle. Another factor that may explain the increased risk for maltreatment demonstrated here is that in substance abusing parents, intoxication or withdrawal may result in impairments such as inconsistencies in the care and education of children and reduced impulse control^35^. Neglect and impairment of consistent care^36^, involvement of children in delusions and inappropriate affects that may cause insecurity in children^37^, may be relevant in mentally ill parents. In addition, there is a risk of an impaired attachment to the child, which is of outstanding importance for the healthy development of children^38,39^: Biological factors such as genetics in the intergenerational transmission of mental illness^40^, altered health behavior such as smoking^2^, and increased exposure to stress followed by changes in stress regulation^41^ may also play a role. The relevance of these factors can hardly be assessed in isolation and can vary depending on the background.

In families affected by mental health problems or substance abuse, these factors often accumulate. Although many parents are aware that they need support, they are often reluctant to accept professional help due to concerns about stigmatization or loss of custody^42^. Importantly, the risk of losing custody of children can be significantly reduced by early intervention^43^—as can the risk of children developing mental disorders by themselves^44^. A higher awareness of the existing risks for children of mentally ill or substance-abusing parents, combined with adequate support services, may thus contribute to the long-term destigmatization of affected parents through prevention.

### Limitations

The assessment of ACEs was based on retrospective self-report, which can lead to underestimation of adverse childhood experiences due to recall bias, shame and misunderstandings^45,46^. This in turn could lead to an underestimation of the results shown. In addition, it was not assessed which household member was mentally ill in the original household or who consumed substances, which substances were abused or which disease was present. Substance abuse and mental illness can be very heterogeneous. Additionally, severity of the individual types of maltreatment, the age at which they were experienced and for how long they were exposed to these experiences was not assessed. The wide age range of our sample is a strength. However, for some outcomes, such as unemployment, this may be a limitation as during the survey, some participants had not yet had the opportunity to be unemployed as they are still at school, for example. Even though missing data is scarce, only valid cases were included into the analysis and selection bias can not be excluded. As this is an observational study, no causality can be derived.

## Conclusion

Against the background of the here presented results, there is an urgent need for greater awareness for the increased risks of children of mentally ill and substance- abusing parents. Affected patients should be systematically asked about minor children and informed about the increased risks for affected children, just as about support offers. The here presented results indicate a need for better care for children of mentally ill and substance-abusing parents. Support for affected families, in which child protection plays a central role, and closer networking of psychiatrists, child and adolescent psychiatrists, paediatricians as well as youth welfare should be recommended.
